# É necessário o uso de aumento com cimento na fixação interna de fraturas trocantéricas em idosos? Revisão sistemática e metanálise

**DOI:** 10.1055/s-0046-1824549

**Published:** 2026-08-14

**Authors:** Adhe Sugandhi, Ni Kadek Saras Dwi Guna, Grace Yulia Alphani Yapson, I Putu Divanaya Suryanov, I Gusti Ngurah Paramartha Wijaya Putra

**Affiliations:** 1 Clínico Geral, Universitas Udayana, Denpasar, Bali, Indonesia; 2 Departamento de Ortopedia e Traumatologia, Ngoerah Hospital, Bali, Indonésia

**Keywords:** cimentos ósseos, fixação interna de fraturas, fraturas do quadril, idoso, bone cements, elderly, fracture fixation, internal, hip fractures

## Abstract

**Objetivo:**

Avaliar a eficácia do aumento com cimento na fixação interna de fraturas trocantéricas em idosos.

**Métodos:**

Uma busca abrangente nas bases de dados MEDLINE, SpringerLink, CENTRAL e Scopus sobre ensaios clínicos randomizados (ECRs) que comparavam a fixação interna com e sem aumento com cimento foi conduzida até fevereiro de 2025. A revisão seguiu as diretrizes da declaração Preferred Reporting Items for Systematic Reviews and Meta-Analyses (PRISMA) de 2020. Modelos de efeitos aleatórios foram utilizados para a combinação das estimativas de efeito. Os desfechos primários foram a falha de fixação e a distância ponta-ápice (DPA). Os desfechos secundários incluíram mortalidade, taxa de reoperação, tempo de internação, perda sanguínea e tempo operatório. O risco de viés foi avaliado com a versão revisada da ferramenta Cochrane Risk of Bias (RoB 2.0; Cochrane Collaboration), e a certeza da evidência foi analisada pelo método Grading of Recommendations, Assessment, Development, and Evaluation (GRADE).

**Resultados:**

Cinco ECRs foram incluídos, num total de 509 pacientes. O aumento com cimento não reduziu significativamente a falha de fixação (risco relativo [RR] = 1,13; IC95% = 0,20–6,43), mas melhorou significativamente a DPA (diferença média [DM] = -2,11 mm; IC95% = -3,13 a -1,09; *p* < 0,0001). Não houve efeito significativo sobre a mortalidade (RR = 1,10; IC95% = 0,54–2,22), nem sobre a taxa de reoperação (RR = 0,12; IC95% = 0,01–2,09). O aumento com cimento reduziu o tempo de internação em 2,5 dias (DM = -2,59; IC95% = -6,87 a 1,69), mas aumentou a perda sanguínea (DM = 49,01; IC95% = -61,78 a 159,8) e o tempo operatório em 11 minutos (DM = 11,8; IC95% = 5,91–17,69).

**Conclusão:**

O aumento com cimento melhora a qualidade da fixação e reduz o tempo de internação em pacientes idosos com fraturas trocantéricas, mas aumenta a perda sanguínea e o tempo operatório. Seu uso deve ser individualizado com base no perfil de risco do paciente.

## Abstract (en)

**Objective:**

To evaluate the efficacy of cement augmentation in internal fixation of trochanteric fractures in the elderly.

**Methods:**

A comprehensive search of the MEDLINE, SpringerLink, CENTRAL, and Scopus databases was conducted up to February 2025 for randomized clinical trials (RCTs) comparing internal fixation with and without cement augmentation. The review adhered to the 2020 Preferred Reporting Items for Systematic Reviews and Meta-Analyses (PRISMA) statement guidelines. Random-effects models were used to pool effect estimates. The primary outcomes were fixation failure and tip-apex distance (TAD). The secondary outcomes included mortality, reoperation rate, length of hospital stay, blood loss, and operative time. The risk of bias was assessed with the revised version of the Cochrane Risk of Bias tool (RoB 2.0; Cochrane Collaboration), and evidence certainty, with the Grading of Recommendations, Assessment, Development, and Evaluation (GRADE) approach.

**Results:**

Five RCTs comprising 509 patients were included. Cement augmentation did not significantly reduce fixation failure (relative risk [RR] = 1.13; 95%CI: 0.20–6.43) but significantly improved the TAD (mean difference [MD] = -2.11 mm; 95%CI: -3.13 to -1.09; *p* < 0.0001). There were no significant effects on mortality (RR = 1.10; 95%CI: 0.54–2.22) or reoperation (RR = 0.12; 95%CI: 0.01–2.09). Cement augmentation shortened the hospital stay by 2.5 days (MD = -2.59; 95%CI: -6.87 to 1.69), but it increased blood loss (MD = 49.01; 95%CI: -61.78 to 159.8) and operative time by 11 minutes (MD = 11.8; 95%CI: 5.91–17.69).

**Conclusions:**

Cement augmentation improves fixation quality and reduces hospitalization in elderly patients with trochanteric fractures, but it increases blood loss and operative time. Its use should be individualized based on patient risk profiles.

## Introdução

As fraturas trocantéricas são um desafio ortopédico comum em idosos, principalmente devido à fragilidade óssea relacionada à idade e à osteoporose. Essas fraturas frequentemente requerem fixação interna; no entanto, os métodos convencionais de fixação podem ser insuficientes em ossos com osteoporose grave, o que leva a complicações como falha de fixação e migração do implante, entre outras. 1 2

Entre os tratamentos de fixação interna para fraturas trocantéricas estão o Gamma3 Nail (Stryker Corporation), o Trochanteric Fixation Nail–Advanced (TFNA; DePuy Synthes Inc.), as hastes de compressão e antirrotação integradas (como a Proximal Femoral Nail Antirotation [PFNA, DePuy Synthes Inc.] e a Intertrochanteric Antegrade Nail [INTERTAN; Smith & Nephew plc]) e o parafuso dinâmico do quadril, ou parafuso deslizante (Sliding/Dynamic Hip Screw [SHS/DHS; DePuy Synthes Inc.]). 3 Cada tipo de fixação interna tem vantagens e desvantagens. As falhas de fixação associadas ao DHS e às hastes cefalomedulares podem chegar a 8,4% e 6,3%, respectivamente. 4 5 Assim, outras técnicas são necessárias para o tratamento, inclusive o aumento com cimento, seja com polimetilmetacrilato (PMMA) ou fosfato de cálcio. 6 7 Embora alguns estudos sugiram a necessidade do aumento com cimento, outros indicam que sua adição não é significativa e pode até aumentar as taxas de complicações, como sangramento. 8 9 10 11 12 Desta forma, mais estudos devem ser realizados para esclarecer definitivamente essa questão.

Este estudo tem como objetivo determinar a necessidade de aumento com cimento na fixação interna de fraturas trocantéricas em idosos, avaliando seu impacto nas falhas de fixação, taxas de cirurgias de revisão e outras complicações.

## Métodos

Esta metanálise seguiu as exigências do *Cochrane Handbook for Systematic Review of Interventions* e foi registrada no International Prospective Register of Systematic Reviews (PROSPERO; CRD420251006740). Por não se tratar de um estudo clínico, não houve necessidade de aprovação ética.

Este estudo incluiu ensaios clínicos randomizados (ECR) sobre aumento com cimento na fixação interna de fraturas trocantéricas em idosos. Os critérios de inclusão foram pacientes maiores de 65 anos com fraturas trocantéricas, fixação interna com TFNA, PFNA, Gamma3 ou SHS/DHS, e acompanhamento mínimo por 6 meses. E os critérios de exclusão foram estudos com cadáveres, revisões, metanálises e relatos de caso, fraturas patológicas, abertas, periprotéticas ou não trocantéricas, e acompanhamento desconhecido, fraturas não traumáticas, ausência de consolidação ou cirurgias de revisão.

### Estratégia de Busca na Literatura

Este estudo foi redigido de acordo com as diretrizes de 2020 da declaração Preferred Reporting Items for Systematic Review and Meta-Analyses (PRISMA). Dois pesquisadores clínicos independentes (AS e NKSDG) realizaram buscas nas bases de dados online MEDLINE, SpringerLink, CENTRAL e Scopus em 24 de fevereiro de 2025. Para minimizar o risco de perda de estudos relevantes, nenhum filtro foi aplicado à estratégia de busca, que é apresentada em detalhes no Material Suplementar.

### Extração de Dados e Avaliação do Risco de Viés

Dois autores (AS e NKSDG) extraíram e registraram os dados relevantes de cada estudo incluído seguindo um protocolo padronizado. As informações extraídas foram autores e ano de publicação, país, período do estudo, dispositivo, tipo de cimento, características demográficas dos pacientes (idade, sexo, tipo de fratura) e duração do acompanhamento. Além disso, a incidência de complicações intraoperatórias e de necessidade de outras cirurgias em cada estudo foi calculada.

Dois autores avaliaram, de forma independente, o risco de viés nos estudos incluídos utilizando a versão revisada da ferramenta Cochrane Risk of Bias (RoB 2.0; Cochrane Collaboration). Quaisquer discrepâncias foram resolvidas por meio de discussão ou, se necessário, consulta a um terceiro avaliador. A ferramenta RoB 2.0 avalia cinco domínios: viés decorrente do processo de randomização, desvios das intervenções pretendidas, ausência de dados de desfecho, mensuração dos desfechos e relato seletivo. Cada domínio é classificado segundo a apresentação de *baixo risco* , *algumas preocupações* ou *alto risco* de viés. Uma avaliação global do risco de viés foi determinada separadamente para cada desfecho de cada estudo, com base nas análises de todos os domínios.

### Desfechos

Os desfechos primários foram a falha de fixação e a distância ponta-ápice (DPA), ao passo que os desfechos secundários incluíram taxas de mortalidade, taxas de reoperação, tempo de internação, perda sanguínea e tempo cirúrgico.

### Análise de Dados

A análise estatística foi realizada no ambiente de desenvolvimento integrado RStudio (grátis e de código livre) para a condução de uma metanálise com o objetivo de avaliar variáveis dicotômicas e contínuas. Para os desfechos dicotômicos (falha de fixação, taxa de mortalidade e taxa de reoperação), foram calculados o risco relativo (RR) com ICs 95%. Para as variáveis contínuas, (DPA, tempo de internação, perda sanguínea e tempo cirúrgico), foi calculada a diferença média (DM) com ICs95%.

A heterogeneidade estatística foi estimada pelo método I ^2^ e pela variância entre estudos (Tau ^2^ ), além da avaliação por meio do teste Q de Cochran. A heterogeneidade também foi avaliada por inspeção visual dos gráficos de floresta; o valor de I ^2^ foi interpretado como baixo (0–40%), moderado (30–60%), substancial (50–90%) e considerável (75–100%). Quando o I ^2^ foi superior a 50%, possíveis fontes de heterogeneidade foram investigadas. O teste Q de Cochran foi utilizado para a determinação da heterogeneidade, com significância estatística definida como *p* < 0,10.

Um modelo de efeitos aleatórios foi aplicado em todas as análises, uma vez que considera a variabilidade entre os estudos. Além disso, realizamos análises de sensibilidade para explorar possíveis fontes de heterogeneidade e avaliar o impacto do viés nas estimativas de efeito. A significância estatística para as análises principais foi definida como *p* < 0,05.

## Resultados

### Resultados da Busca

No total, 512 referências foram identificadas e avaliadas. A Fig. ilustra o processo de busca e seleção. Após a remoção de registros duplicados e daqueles considerados inelegíveis por ferramentas automatizadas, 388 registros foram submetidos à triagem por título e resumo, dos quais 352 foram excluídos por não serem relevantes. Ao todo, 36 artigos com texto completo foram avaliados quanto à elegibilidade e, em seguida, 31 foram excluídos devido a delineamentos experimentais inadequados, idioma diferente do inglês, população de pacientes incorreta ou desfechos diferentes. Ao final, cinco ECRs 8 9 10 11 12 atenderam aos critérios de inclusão e integraram a síntese qualitativa e quantitativa (metanálise).

### Características dos Estudos

As pesquisas foram realizadas em 4 bases de dados, levando à inclusão de 5 estudos 8 9 10 11 12 (1997–2019) conduzidos na Suécia, na Itália, na Alemanha, na China e na Colômbia. Estes estudos avaliaram o aumento com cimento na fixação de fraturas do fêmur proximal utilizando DHS, Gamma3, PFNA, TFNA e a Aimstrong Proximal Femoral Nail (APFN, Double Medical Technology Inc.). Como resumido na Tabela 1 , o PMMA foi utilizado em 3 estudos, 8 10 11 o fosfato de cálcio, em um 12 e, no estudo restante, 9 o material não foi relatado. Os volumes de cimento variaram de 2 a 20 mL; 2 estudos 9 10 não relataram esses valores.

**Tabela 1:** Características dos estudos incluídos

Autor (ano)	País	Período do estudo	Dispositivo	Tipo de cimento	Cimento injetado	Tamanho total da amostra		Sexo (F/M)		Idade média (anos)		AO31-(A1/A2/A3) AO32A2		Acompanhamento
						A	C	A	C	A	C	A	C	
Mattsson et al. 12 (2005)	Suécia	1997–2000	DHS	Fosfato de cálcio	20 mL	55	57	44/11	47/10	81,2 ± 7,0	82,0 ± 6,3	NR	NR	6 meses
Dall'Oca et al. 11 (2010)	Itália	1997–2000	Gamma3 nail	PMMA	2–3 mL	40	40	26/14	30/10	85,3 ± 2,3	82,3 ± 1,2	0/20/15	0/22/14	12 meses
Kammerlander et al. 10 (2018)	Alemanha	Janeiro de 2006–março de 2010	PFNA	PMMA	NR	87	135	99/19	86/21	86,1 ± 4,6	85,6 ± 4,9	0/9/9	0/96/22	12 meses
Ni et al. 9 (2022)	China	Março de 2012–julho 2015	PFNA e APFN	NR	NR	22	20	NR	NR	NR	NR	NR	NR	12 a 16 meses
Salazar et al. 8 (2023)	Colômbia	Janeiro de 2017–janeiro 2019	TFNA	PMMA	4 mL	23	30	19/4	23/7	83,87	85,91	5/15/2/1	8/7/12/3	12 meses

**Abreviaturas:** A, aumento; APFN, Aimstrong Proximal Femoral Nail; C, controle; DHS, Dynamic Hip Screw); F, feminino; M, masculino; NR, não relatado(a); PFNA, Proximal Femoral Nail Antirotation; PMMA, polimetilmetacrilato; TFNA, Trochanteric Fixation Nail–Advanced.

O número de participantes foi de 22 a 87 nos grupos com aumento e de 20 a 135 nos grupos de controle. Em sua maioria, os participantes eram mulheres idosas (idade média: 81,2–86,1 anos nos grupos com aumento e 82,0–85,9 nos grupos de controle). Três estudos 8 10 11 relataram as classificações da Arbeitsgemeinschaft für Osteosynthesefragen (AO; principalmente AO31-A2/A3). De modo geral, o acompanhamento ocorreu por 12 meses; em um estudo, 12 foi de 6 meses, e em outro, 9 de 12 a 16 meses.

### Avaliação do Risco de Viés

Dois autores (AS e IPDS) avaliaram independentemente o risco de viés para os desfechos de falha de fixação e DPA utilizando a ferramenta RoB 2.0 ( Fig. 2 ). Três estudos incluídos apresentaram baixo risco de viés ou algumas preocupações, ao passo que dois 9 10 tinham alto risco global de viés, principalmente relacionados a desvios das intervenções pretendidas e à mensuração dos desfechos. O viés decorrente do processo de randomização e da ausência de dados de desfecho foi, em geral, baixo. Como comumente observado em estudos cirúrgicos, os domínios relacionados ao cegamento representaram as principais fontes potenciais de viés; no entanto, os desfechos primários (falha de fixação e DPA) são medidas objetivas e, portanto, menos suscetíveis a vieses de desempenho e detecção. A presença de estudos com alto risco de viés e amostras pequenas pode ter contribuído para a imprecisão em vários desfechos; logo, a certeza global da evidência deve ser interpretada com cautela.

**Fig. 1:**
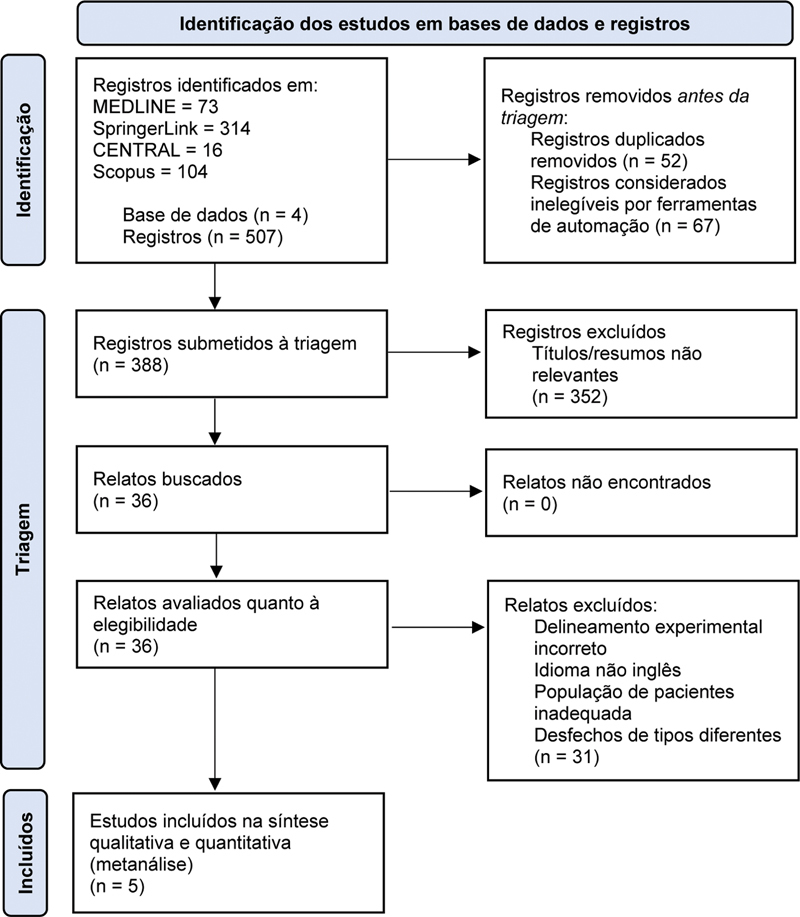
Diagrama de fluxo dos resultados da busca na literatura segundo a declaração Preferred Reporting Items for Systematic Reviews and Meta-Analyses (PRISMA) de 2020.

**Fig. 2:**
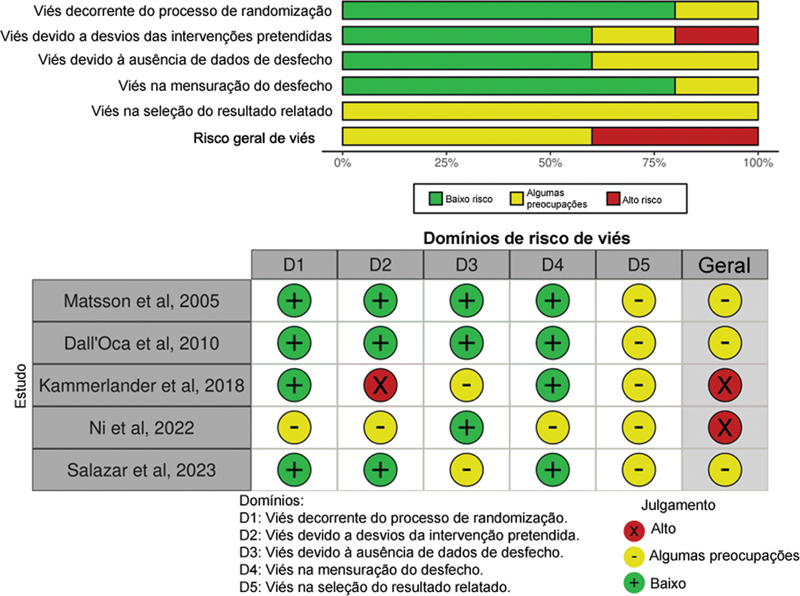
Risco de viés quanto ao desfecho.

### Resultados da Metanálise

A comparação dos desfechos do aumento com cimento na fixação interna de fraturas trocantéricas está resumida na Tabela 2 e na **Tabela S3** do **Material Suplementar** ).

**Tabela 2:** Resumo dos achados

Desfecho	Efeitos absolutos estimados (IC95%)	Efeito relativo (IC95%)	Número de participantes (estudos)	Certeza da evidência (GRADE)	Comentários
Falha de fixação	2 a mais por 1.000 (de 14 a menos a 98 a mais)	RR = 1,13 (0,20–6,43)	387 (3 ECRs)	Baixa ^a,b^	Muito incerto quanto ao efeito sobre a falha de fixação
Distância ponta–ápice	DM = 2,11 mm menor (3,13–1,09 menor)	–	245 (3 ECRs)	Alta ^a^	Provável redução da DPA após o aumento com cimento
Mortalidade (1 ano)	9 a mais por 1.000 (de 42 a menos a 112 a mais)	RR = 1,10 (0,54 a 2,22)	302 (2 ECRs)	Baixa ^a,b^	Diferença pequena ou nula na mortalidade
Reoperação	39 a menos por 1.000 (de 44 a menos a 48 a menos)	RR = 0,12 (0,01 a 2,09)	222 (2 ECRs)	Baixa ^a^	O aumento com cimento pode reduzir a taxa de reoperação
Tempo de internação	DM = 2,59 dias menor (6,87 menor a 1,69 menor)	–	234 (3 ECRs)	Baixa ^a,c^	O aumento com cimento pode causar uma pequena redução no tempo de internação
Perda sanguínea	DM = 49,01 mL maior (61,78 menor a 159,8 maior)	–	154 (3 ECRs)	Baixa ^a,c^	Evidência incerta quanto à diferença na perda sanguínea
Tempo cirúrgico	DM = 11,8 min maior (5,91–17,69 maior)	–	234 (3 ECRs)	Moderada ^a,c^	O aumento com cimento aumenta o tempo cirúrgico

**Abreviaturas:** DM, diferença média; DPA, distância ponta-ápice; ECR, ensaio clínico randomizado; GRADE, Grading of Recommendations, Assessment, Development, and Evaluation; min, minutos; RR, risco relativo.

**Notas:**^a^ Não houve cegamento de participantes e da equipe; ^b^ baixa robustez; e ^c^ alta heterogeneidade.

#### Desfechos Primários

##### Falha de Fixação

**Fig. 3:**
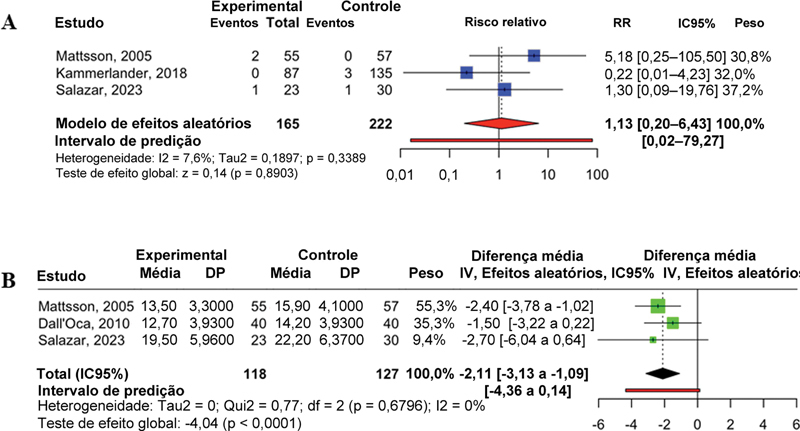
Gráfico de floresta de comparação dos desfechos primários: ( **A** ) falha de fixação; e ( **B** ) distância ponta-ápice. **Abreviaturas:** df, *degrees of freedom* (graus de liberdade); DP, desvio padrão; IV, *inverse variance* (variância inversa); RR, risco relativo.

Dados de três estudos, 8 10 12 com um total de 387 participantes, foram combinados nesta metanálise para a avaliação das falhas de fixação. No grupo submetido ao aumento com cimento, foi relatado um caso de *cut-through* , 8 além de dois casos de afrouxamento da placa. 12 A análise combinada não demonstrou benefício significativo do aumento com cimento, com IC95% amplo (RR = 1,13; IC95% = 0,20–6,43; *p* = 0,8903). A certeza da evidência foi classificada como baixa. Além disso, não houve indicação de heterogeneidade estatística substancial (Tau ^2^ = 0,1897; I ^2^ = 7,6%) ( Fig. 3A ; **Material Suplementar, Fig. S1** ).

##### Distância Ponta-Ápice

Três estudos 8 11 12 (245 participantes) relataram dados sobre a DPA. A análise demonstrou que o grupo submetido ao aumento com cimento apresentou DPA significativamente menor em comparação ao grupo sem aumento, com DM de -2,11 mm (IC95% = -3,13 a -1,09; *p* < 0,0001). Isso indica que o aumento com cimento pode melhorar a precisão do posicionamento do implante. Não houve heterogeneidade entre os estudos incluídos (Tau ^2^ = 0; I ^2^ = 0%; *p* = 0,6796) ( Fig. 3B ; **Material Suplementar, Fig. S2** ).

#### Desfechos Secundários

##### Mortalidade

**Fig. 4:**
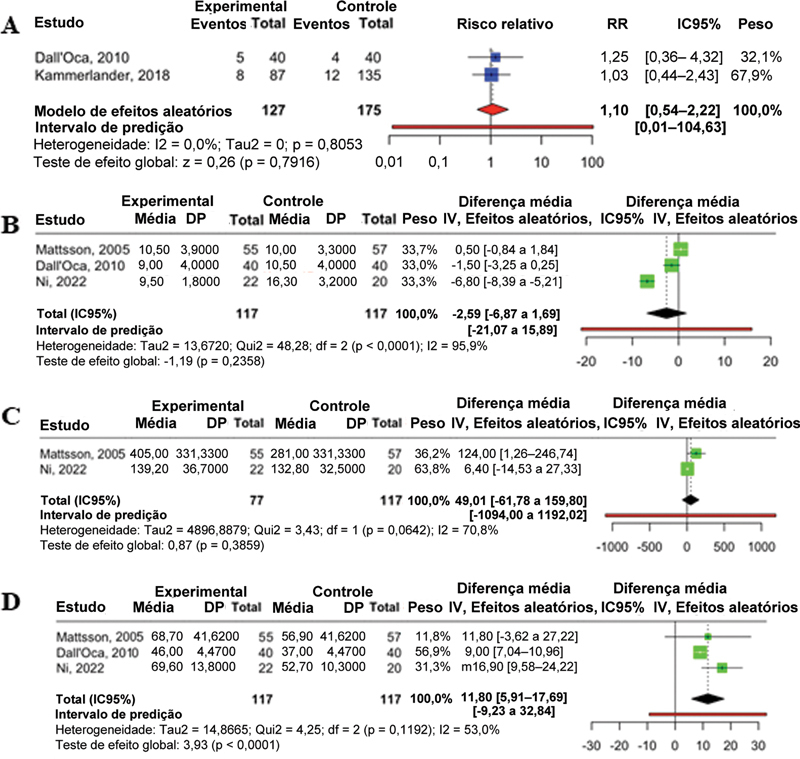
Gráfico de floresta de comparação dos desfechos secundários: ( **A** ) mortalidade; ( **B** ) tempo de internação; ( **C** ) perda sanguínea; e ( **D** ) tempo operatório. **Abreviaturas:** df, *degrees of freedom* (graus de liberdade); DP, desvio padrão; IV, *inverse variance* (variância inversa); RR, risco relativo.

Dois estudos 10 11 apresentaram dados sobre mortalidade em 1 ano, os quais foram combinados para facilitar a análise. A taxa de mortalidade no grupo submetido ao aumento com cimento foi de 10,2%, em comparação a 9,1% no grupo sem aumento. No entanto, não houve diferença estatisticamente significativa na mortalidade pós-operatória entre os dois grupos (RR = 1,10; IC 95% = 0,54–2,22; *p* = 0,7916; I ^2^ = 0%) ( Fig. 4A ).

##### Taxa de Reoperação

A taxa de reoperação foi relatada em dois estudos. 10 12 Porém, somente um estudo 10 mostrou esse desfecho separadamente nos dois grupos de tratamento, que não apresentaram diferença significativa entre si (RR = 0,12; IC95% = 0,01–2,09; *p* = 0,1454) ( Fig. 4B ); além disso, a evidência foi considerada de baixa qualidade. Nesse caso, a heterogeneidade não foi aplicável.

##### Tempo de Internação

Dados de três estudos 9 11 12 (com 117 participantes em cada grupo) foram incluídos na metanálise para comparar o tempo de internação hospitalar entre o grupo submetido ao aumento com cimento e o grupo de controle. Os resultados demonstraram DM de -2,59 dias a favor do grupo submetido ao aumento com cimento; no entanto, essa diferença não foi estatisticamente significativa (DM = -2,59; IC95% = -6,87 a 1,69; *p* = 0,2358). O intervalo de predição foi bastante amplo, de -21,07 a 15,89, o que indica considerável variabilidade no efeito verdadeiro entre diferentes contextos. A heterogeneidade estatística foi substancial (Tau ^2^ = 13,6720; I ^2^ = 95,9%; Qui ^2^ = 48,28; graus de liberdade [ *degrees of freedom* , df, em inglês] = 2; *p* < 0,0001) ( Fig. 4B ).

##### Perda Sanguínea

Dois estudos 9 12 (com 77 participantes por grupo) relataram perda sanguínea intraoperatória. Os resultados combinados demonstraram uma DM de 49,01 mL a favor do grupo de controle; no entanto, essa diferença não foi estatisticamente significativa (DM = 49,01, IC95% = -61,78 a 159,80; *p* = 0,3859). O intervalo de predição foi amplo (de -1094,00 a 1192,02), o que indica alto grau de incerteza quanto ao efeito verdadeiro em diferentes contextos. A heterogeneidade entre os estudos incluídos foi de moderada a substancial (Tau ^2^ = 4896,8879; I ^2^ = 70,8%; Qui ^2^ = 3,43; df = 1; *p* = 0,0642) ( Fig. 4C ).

##### Tempo Operatório

Três estudos 9 11 12 (com 117 participantes por grupo) relataram o tempo operatório. A análise combinada demonstrou tempo operatório significativamente maior no grupo submetido ao aumento com cimento em comparação ao grupo sem aumento, com DM de 11,80 minutos (IC95% = 5,91–17,69; *p* < 0,0001). O intervalo de predição variou de -9,23 a 32,84, o que sugere alguma variabilidade no efeito verdadeiro entre diferentes contextos. A heterogeneidade foi moderada (Tau ^2^ = 14,8685; I ^2^ = 53,0%), embora não tenha sido estatisticamente significativa (Qui ^2^ = 4,25; df = 2; *p* = 0,1192) ( Fig. 4D ).

### Viés de Publicação

Devido ao pequeno número de estudos incluídos ^8–12^ (n = 5), não foi realizada uma avaliação formal do viés de publicação por meio da visualização de gráficos em funil ou testes estatísticos, como os de Egger ou Begg. De acordo com as recomendações do *Cochrane Handbook* , são necessários pelo menos 10 estudos para detectar adequadamente qualquer assimetria e avaliar o viés de publicação. Com menos estudos, gráficos em funil e métodos baseados em regressão apresentam poder insuficiente e podem levar a interpretações enganosas. Portanto, como o viés de publicação não pode ser formalmente avaliado nesta análise, trata-se de uma possível limitação dos achados deste estudo.

### Análise de Sensibilidade

**Fig. 5:**
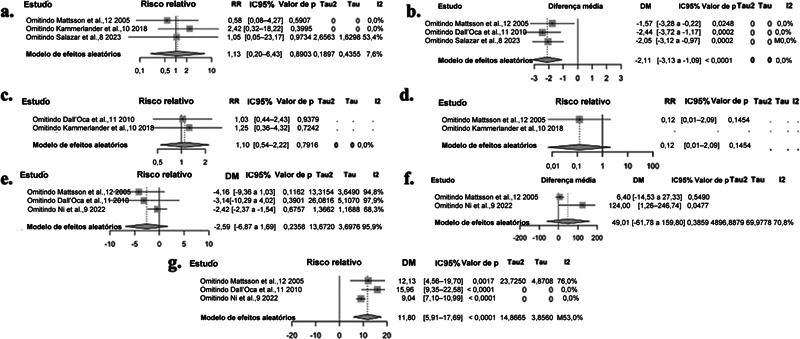
Análise *leave-one-out* : ( **A** ) falha de fixação; ( **B** ) distância ponta-ápice; ( **C** ) mortalidade; ( **D** ) reoperação; ( **E** ) tempo de internação; ( **F** ) perda sanguínea; e ( **G** ) tempo operatório. **Abreviaturas:** DM, diferença média; RR, risco relativo.

Uma análise de sensibilidade utilizando a abordagem *leave-one-out* foi realizada para determinar a robustez das estimativas combinadas em todos os desfechos ( Fig. 5 ). A análise demonstrou que a redução significativa da DPA (DM = -2,11; IC95% = -3,13 a -1,09; *p* < 0,0001) e o aumento do tempo operatório (DM = 11,80; IC95% = 5,91–17,69; *p* < 0,0001) permaneceram estáveis, o que indica achados consistentes independentemente de qual estudo fosse excluído. Por outro lado, desfechos como falha de fixação (RR = 1,13; IC95% = 0,20–6,43; *p* = 0,89), taxa de mortalidade (RR = 1,10; IC95% = 0,54–2,22; *p* = 0,79), taxa de reoperação (RR = 0,12; IC95% = 0,01–2,09; *p* = 0,15), tempo de internação (DM = -2,59; IC95% = -6,87 a 1,69; *p* = 0,24) e perda sanguínea (DM = 49,01; IC95% = -61,78 a 159,80; *p* = 0,39) não apresentaram efeitos estatisticamente significativos e variaram quanto à heterogeneidade; além disso, tempo de internação e a perda sanguínea foram bastante variáveis entre os estudos (I ^2^ = 95,9% e 70,8%, respectivamente). Esses achados sugerem que, embora certos parâmetros operatórios, como DPA e tempo operatório, sejam afetados de forma consistente pela intervenção, outros desfechos podem ser influenciados por fatores específicos dos estudos ou não apresentar poder suficiente para detecção de diferença significativa.

## Discussão

Esta metanálise demonstrou que o aumento com cimento na fixação interna de fraturas trocantéricas melhorou significativamente o posicionamento do implante, conforme indicado pela redução da DPA. No entanto, não houve benefício estatisticamente significativo na redução da falha de fixação, reoperação ou mortalidade. Assim, embora a hipótese de estabilização mecânica encontre apoio parcial, a hipótese geral de que o aumento com cimento melhora os desfechos clínicos só é parcialmente corroborada.

A melhora estatisticamente significativa na DPA reflete um benefício mecânico que pode favorecer a melhor ancoragem do implante. No entanto, isso não se traduziu em diferenças clinicamente relevantes em desfechos-chave, como taxas de reoperação ou tempo de internação, que não apresentaram alterações significativas. A diferença no tempo operatório foi clinicamente modesta, porém estatisticamente significativa. Assim, o benefício mecânico do aumento pode ser clinicamente relevante somente em alguns casos de alto risco com fraturas instáveis ou baixa qualidade óssea.

Em comparação a estudos observacionais e biomecânicos prévios, que demonstraram benefícios mais definitivos do aumento com cimento na prevenção da migração do implante e *cut-out* , nossa análise de ECRs gerou conclusões mais cautelosas. Embora estudos como os conduzidos por Kammerlander et al. 10 e Salazar et al. 8 sugiram melhora na estabilidade do construto, não demonstraram de forma consistente melhora nos desfechos quando submetidos a uma metanálise rigorosa. Isso evidencia uma lacuna entre as vantagens teóricas/mecânicas e a efetividade clínica mensurável.

A força desta revisão reside na adesão às diretrizes PRISMA, na metodologia robusta e na análise de sensibilidade. No entanto, as conclusões são limitadas pelo pequeno número de ECRs de alta qualidade e pela heterogeneidade entre eles. Pesquisas futuras devem se centrar na estratificação dos pacientes com base na estabilidade da fratura e na qualidade óssea, utilizando medidas de desfecho padronizadas, como escores de mobilidade funcional, e explorando a relação custo-benefício para direcionar a aplicação clínica.

## Conclusão

O aumento com cimento na fixação interna de fraturas trocantéricas reduziu significativamente a DPA, sustentando melhor o posicionamento do implante. No entanto, não reduziu de forma significativa a falha de fixação, as taxas de reoperação ou a mortalidade em 1 ano. Da mesma forma, não foram observadas diferenças estatisticamente significativas no tempo de internação, na perda sanguínea intraoperatória ou na frequência global de reoperação. O tempo cirúrgico foi significativamente maior no grupo com aumento. Com base nesses achados, a hipótese de que o aumento com cimento melhora os desfechos clínicos além do posicionamento mecânico não se sustenta.
